# A scoping review of admission criteria and selection methods in nursing education

**DOI:** 10.1186/s12912-020-00510-1

**Published:** 2020-12-14

**Authors:** Vahid Zamanzadeh, Akram Ghahramanian, Leila Valizadeh, Farzaneh Bagheriyeh, Marita Lynagh

**Affiliations:** 1 Department of Medical Surgical Nursing, School of Nursing and Midwifery, Tabriz University of Medical Sciences, Tabriz, Iran; 2 Department of Pediatric Nursing, School of Nursing and Midwifery, Tabriz University of Medical Sciences, Tabriz, Iran; 3 School of Medicine & Public Health, University of Newcastle, Hunter Medical Research Institute (HMRI), Newcastle, Australia

**Keywords:** Admission criteria, Selection methods, Nursing student selection, Nursing education

## Abstract

**Background:**

Nursing education institutions are required to select and train applicants who have appropriate characteristics for delivering effective healthcare. Unlike other healthcare professions and despite the need to attract and select a competent workforce, there has been no comprehensive analysis of the selection criteria and methods used to recruit nursing students. As there is relatively limited prior research available, we conducted a scoping review to explore and synthesise the existing evidence regarding admission criteria and selection methods of nursing students and for the purpose of identifying an agenda for future research in this field.

**Methods:**

Our scoping review follows the Arksey and O’Malley five-step proposition including identifying the research question and relevant studies, study selection, tabulation of data, and summarizing and reporting the results. Seven databases (PubMed, CINAHL, Scopus, ERIC, SID, Irandoc and PsycINFO) were searched systematically using relevant keywords. Articles on admission of undergraduate nursing students published in both English and/or Persian from 2006 to 2019 were retrieved.

**Results:**

Existing research evidence suggests that nursing students are largely selected on the basis of two criteria - “cognitive-academic abilities” and “non-cognitive abilities.” Cognitive-academic abilities were assessed in four main dimensions of mathematics, language, natural sciences and reasoning skills mainly through standardized tests and academic records. Our review shows a wide range of non-cognitive characteristics are evaluated in nursing applicants including: morality, interpersonal communication skills and psychological strength. The selection method most commonly used to assess characteristics was through interviews (panel interviews or multiple mini interviews). Other methods included references, personal statements and personality assessment tools.

**Conclusions:**

This is the first scoping review of literature regarding nursing education selection and recruitment. Results can be used to inform nursing education policymakers and institutions in the design of their selection practices. Future research should concentrate on the evaluation and improvement methods of student selection including content and predictive validity analysis of multiple mini interview and standardized tests, development of cost-effective selection methods and job analysis studies to identify specific non-cognitive characteristics for nursing.

## Background

Student selection in the health professions is increasingly being recognised as an important issue [1]. The ultimate goal of student selection is to identify who will go on to be the most effective clinicians in delivering patient care, which ultimately relates to positive health outcomes [2]. Selection of students who can successfully complete their education and have necessary professional qualifications is currently considered a main challenge of health education institutions in the world [3].

Nurses, who play a key role in promoting individual and community health [4], comprise the largest group of health care workforce [5] with approximately 35 million nurses and midwives worldwide. Choosing the right student for the nursing profession will ensure job compatibility, improves nursing workforce performance in the future and ensures the safety and well-being of patients [6]. Additionally it maximizes the effectiveness of health systems and can ultimately lead to improved nursing care. It also helps to better the public image of the nursing profession in the society [7].

Recently, the number of nursing program applications has increased both internationally and in Iran [8, 9]. One of the major challenges in the nursing education is selecting competent applicants who are most likely to accomplish the training program successfully, and make a long-term effective contribution to their profession, the general public, and the community [10]. This issue has received much attention in recent years, largely due to growing concerns of diminishing quality of nursing care, high attrition rates, limited resources and students’ academic failure [11–13]. In addition, nursing instructors and educators [14] have reported a rise in unprofessional attitudes and behaviours of nursing students, further demonstrating the need for the assessment of the professional skills of applicants to nursing, in addition to academic performance [7].

### Selection for nursing education in Iran

Selection methods for entering the nursing profession is considered a key nursing challenge in Iran. Currently, the fit between nursing applicants’ personal characteristics and requirements of the nursing profession are not considered. This has reduced the efficiency of nurses’ performance and impeded the development and maintenance of a sustained, efficient workforce [15, 16]. Since the 1980s, the only criterion utilised in Iran has been success in the University Entrance Exam, which takes the format of a multiple choice written test [17]. A large number of graduated from high school sit the entrance exam annually and admit different majors based on their ranks in this exam [18]. This exam caters for all majors, and hence it cannot take specific features and perquisites for each profession into account [19], where arguably criteria for the health professions may be different to other disciplines and professions.

Several obstacles have impacted the nursing student admission system and nursing profession in Iran in recent years. A significant number of high school graduates admitted to nursing schools through the current system leave before completion because of the mismatch between their personal traits and those required by the nursing profession or they lack sufficient motivation to become qualified nurses [20]. Another important negative effect is reduced efficiency and effectiveness of nurses in their job duties, which is often attributed to sub-optimal selection. In most cases, failure of individuals to effectively perform their job in the organization arises from inconsistency of their psychological characteristics with the job they are undertaking rather than the lack of technical skills or intelligence [21]. This can lead to reduced satisfaction, job failure [22], increased job burnout, decreased performance [21] and reduction of nursing care quality [23].

Nursing education institutions are responsible for selecting and training applicants who have the characteristics necessary for developing and transforming the future of the nursing profession [24, 25]. They are required to have clear admission policies relating to the selection process and minimum admission criteria [26]. However, there is a Lack of information based on research evidence regarding nursing students’ admission criteria and selection practices. Given this knowledge gap and the importance of selecting the right candidates for entry into the nursing profession, a comprehensive analysis of existing research on admission criteria and selection methods of undergraduate nursing students was conducted.

### Objectives and review questions

This study aimed to review existing research evidence regarding nursing students’ selection criteria and selection methods. The research questions were:

1. What criteria are being used to select applicants?
2. Which selection methods are being used to assess applicants as part of selection into undergraduate nursing students?
3. What does the evidence show regarding the predictive validity of selection methods with students’ academic performance?

## Method

### Study design

This scoping review was conducted based on the PRISMA guidelines (see the supplementary data 1) [27, 28]. The five steps included: identifying the research questions; identifying relevant studies; study selection; tabulation of data; and collating, summarizing and reporting the results [29].

### Search strategy

Systematic searches were conducted in databases from April to August 2019 by two researchers. Preliminary searches on PubMed and CINAHL for student selection criteria and methods were performed using the keywords “criteria”, “selection methods”, “nursing school”, “admission criteria” and “nursing student.” The title and abstract of articles were reviewed and new keywords were identified for the full article search. The final search was performed using the following keywords in PubMed, SID, Irandoc, CINAHL, Scopus, ERIC and PsycINFO databases using the Boolean operators “OR” and “AND”:

- “Criteria” OR “cognitive” OR “Non cognitive” OR “admission criteria”
- “nursing student” OR “nursing application” OR “nursing education” OR nursing candidate
- “selection” OR “admission” OR “entry” OR “entrance” OR “recruitment” OR “prerequisite”
- “selection methods” OR “Selection process”
- “test” OR “interview” OR “predictive” OR “psychometric” OR “personality” OR “emotional intelligence” OR “aptitude test” OR “academic record” OR “academic attainment” OR “performance” OR “success”

Search for Persian Literature had no result. The references of the selected articles were also searched manually.

### Study selection

**Fig. 1:**
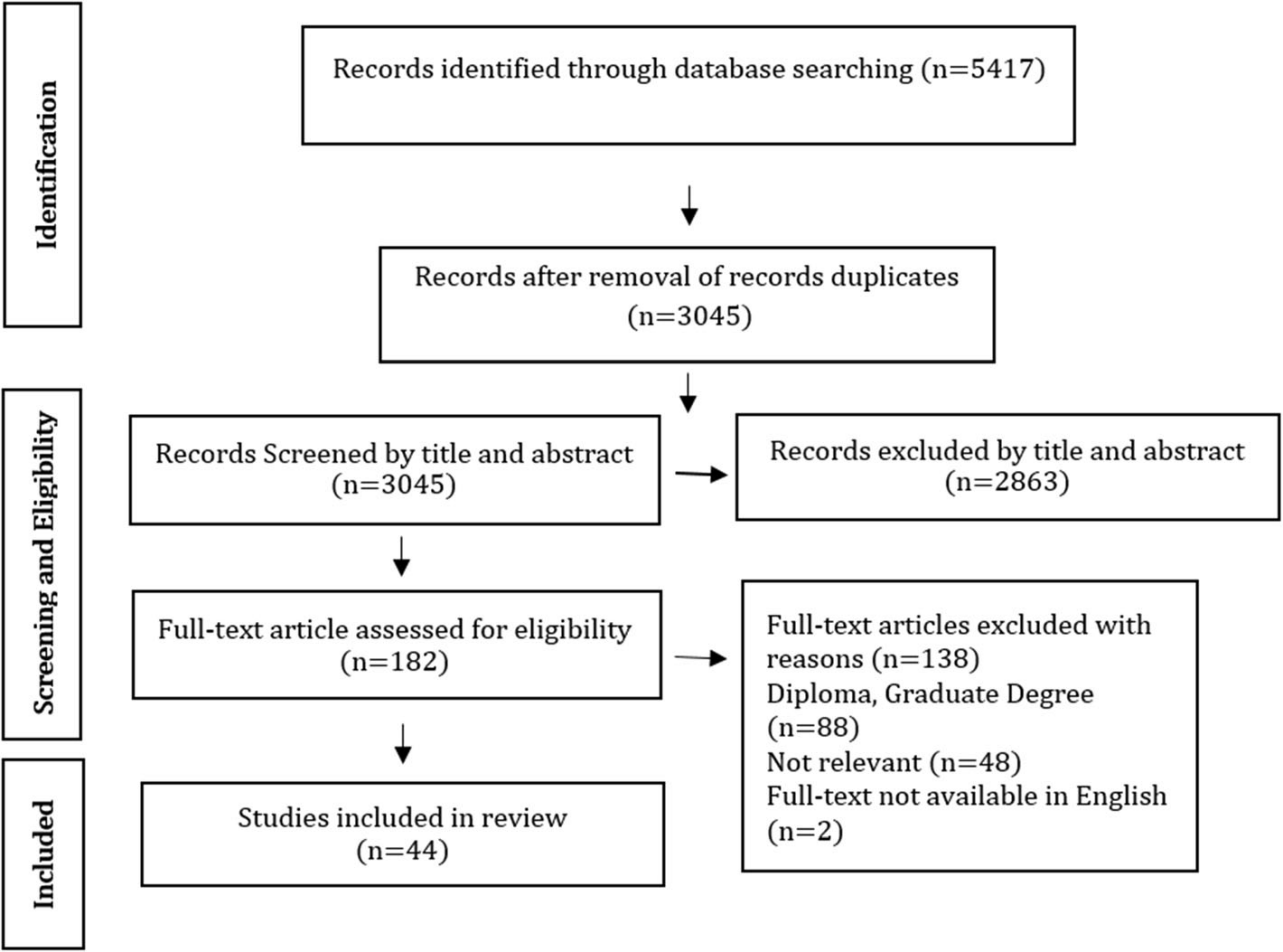
Flow diagram of study selection

Studies were selected according to inclusion and exclusion criteria. Inclusion criteria were Persian and English articles on admission of undergraduate nursing students published from 2006 to 2019. Commentaries, editorials and opinion papers were excluded. The title, abstract and full text of the articles was reviewed by four researchers (VZ, AG, LV AND FB) according to the inclusion and exclusion criteria. Any disagreements resolved by discussion and consensus with the research team. The flow diagram for the article selection process is summarized in Fig. 1.

### Data extraction

Key information extracted from included articles included the author, year, country, main purpose, participants, study design and main results by two reviewers. The data chart was performed independently by two reviewers and then the results were discussed. Data charting was continuously updated in an iterative process (Table 1). The extracted data then were analyzed and interpreted.

**Table 1:** Study characteristics of included articles (*N* = 44)

Author, year, Country, article type	Purpose	Participants	Design	Main study findings
Stuenkel 2006. USA [30]. Research article	To explore the predictive value of various standardized examinations and achievement measures for NCLEX (National Council Licensure Examination-Registered Nurse) performance.	312 BSN students from 6 graduating classes who took the NCLEX for the first time (1997–2001).	Correlational design	The entrance criteria variables of GPA, NLN Pretest, and SAT total scores accounted for 51% of the variation (pass/fail status in NCLEX-RN) and identified 67% (10) of the fail group correctly. The results of this study suggested that entry-level predictors are related to NCLEX success. However, prerequisite GPA alone was not a good predictor.
Newton et al. 2007, USA [31]. Research article	To explore predictive value of scholastic and nursing aptitude of early academic achievement in a BSN (Bachelor of Science in Nursing) program	164 sophomore nursing students.	Exploratory descriptive design	Scholastic and nursing aptitude together predicted 20.2% of the variance in early academic achievement, scholastic aptitude only 15.4% of the variance. Preadmission GPA was more important predictor of 1st semester GPA than TEAS-scores.
Hayes 2007, Canada [32]. Research article	A qualitative descriptive study designed to explore the nature of recruitment practices for basic baccalaureate degree nursing programs in Ontario	15 interviews of nursing faculty and institutional liaison officers, and relevant database materials	Qualitative descriptive design	Supplementary selection methods such as Interview, reference letters, autobiographies (personal statements) are necessary Minimum grade set as a requirement in ac-academic achievement.
Whitehead et al. 2007, UK [33]. Research article	To identify of factors necessary for recruitment and selection of nursing students	106 students from three secondary schools	mixed methods	Examine applicants’ personal characteristics in the selection process (caring, good communication skills, helpful, patient, friendly, understanding and supportive, good social skills, kind, determination/physically strong, trustworthy, considerate, able to give advice, reliable, able to stand the sight of blood, considerate, altruistic, responsible, able to cope with death, open-minded.
Ahmad & Safadi. 2009, Amman [14]. Research article	to examine Relationship between GPA and desire to study nursing with the Chance of Success in Nursing	224 nursing students	A cross-sectional design	School grades and students’ desire to study nursing are recommended as an admission criteria for potential success in nursing programs the choice to study nursing based on desire was able to predict that students will be more satisfied with studying nursing
Newton and Moore 2009, USA [34] Research article	To describe the relationships among scholastic aptitude, nursing aptitude, BSN student attrition prior to the final semester, and BSN student readiness for the NCLEX-RN.	94 BSN students.	Exploratory descriptive design	The final model indicated that scholastic aptitude was predictive of NCLEX-RN readiness but nursing aptitude was not. Neither scholastic nor nursing aptitude predicted student attrition.
McGahee et al. 2010, USA [35]. Research article	To examine student academic variables from a BSN nursing program to determine factors predicting success in NCLEX-RN.	153 graduates of BSN nursing programs over a period of 3 years between fall 2006 and spring 2009.	Retrospective correlational design	Science GPA (prior to admission, incl. Anatomy, Physiology, Chemistry) predicts success in NCLEX-RN test.
Wolkowitz & Kelley 2010. USA [36] Research article	To determine the strength of TEAS sub scores (science, math, reading, English) in predicting early nursing success.	4105 RN students	correlational design	Strongest predictor of early BSN nursing program success was science subtest, followed by reading, written/verbal, and mathematics. 14.9% of the variance in predicting early nursing program success was explained by the science sub score alone.
Timer & Clauson. 2011, Canada [8] Research article	Does the admission process give reliable, valid and fair method of predicting students’ succession in regard to under-graduate academic and clinical courses and also the GPA?	249 students admitted to a Canadian accelerated baccalau-reate nursing program over a 4 year study period.	Retrospective correlational design	Among the selection methods, only academic records were able to predict students’ academic success.
Schmidt & MacWilliams. 2011, USA [37]. Review article	A systematic review of mostly used admission criteria for prelicensure nursing programs and the relationship between these criteria and success in nursing undergraduate pro-grams.	Review from different articles.	systematic review	GPA of courses presumed to be essential in Nursing (English, psychology and sciences) Achievement in sciences (biology, psychology, pathophysiology) in predicting success in nursing programs. Standardized tests used in pre admission to nursing programs. Use of Personal interviews to explore personal characteristics and the important consequence of reducing the rate of attrition Motivational essays. Nursing education outcomes Prior experience in healthcare, volunteerism and other services as a selection method tool.
Shulruf et al. 2011, New Zealand [38]. Research article	The study focused on and high-lighted the predictive value of Undergraduate Grade Point Average as the best predictors for student achievements in their first year in undergraduate program	134 students in the undergraduate nursing program in the University of Auckland	Retrospective correlational design	The best predictor for the first year GPA is the National Certificate of Educational Achievement Grade Point Average. (NCEAGPA). The next best predictor is the university admission ranking scores. The NCEA is the secondary school assessment system in New Zealand.
Hernandez 2011. USA [39]. Doctoral dissertation	To examine the relationships between quantifiable cognitive preadmission variables and BSN program outcomes.	275 nursing students.	Longitudinal design	TEAS composite and section scores correlated with the study outcomes more strongly than GPA. TEAS composite and Science section were especially strong predictors of student success. TEAS composite score is strongly related to Fundamentals test benchmarking midway through the nursing program. Student withdrawal is significantly correlated with the TEAS Composite score.
Dante et al. 2011, Australia [40]. Research article	To define the factors associated with academic success or failure.	117 nursing students enrolled in years 2004–05 on two different bachelor’s courses.	Retrospective correlational design	Having good entry exam scores was associated with academic success.
Grossbach & Kuncel 2011, USA [41]. Research article	To examine the power of key admission and nursing school variables for predicting NCLEX-RN.	7159 participants yielded correlation estimates for 13 different predictors	meta-analysis	SAT and ACT predicted passing the NCLEX-RN. Prenursing (GPA) was also predictive, but to a lesser extent.
Pitt et al. 2012, Australia [42] Review article	To identify factors that influence preregistration nursing students’ academic performance, clinical performance and attrition.	44 articles	integrative review	The most important influencing factors include: demographic characteristics, academic status, cognitive and personality / behavioral factors.
Jarmulowicz 2012, USA [43]. Doctoral dissertation	To examine the admission requirements of nursing programs to better understand the philosophical underpinnings	13 BSN student handbooks and academic bulletins, extraction of admission criteria. 33 full-time teachers	Descriptive correlational design	35 admission criteria were used by nurse education programs. All education programs shared dual admission process (university admission followed by nursing program admission) and high school transcripts. Admission criteria for baccalaureate degree programs ranged from eight to 13 criteria
Herrera 2012, USA [44] Doctoral dissertation	To understand the patterns of selection, preparation, retention and graduation of undergraduate pre-licensure clinical nursing students	584 nursing students enrolled in 2007 and in 2008	Design not stated	Prerequisite courses of Human Nutrition, Clinical Healthcare Ethics, and Human Pathophysiology were predictive of completing the program in the four terms. NET scores did not predict program completion.
Rodgers et al. 2013, UK [45]. Research article	Identification of best practices in recruitment, selection and retention across Scottish universities providing pre-registration programs.	10 universities	qualitative descriptive design	GPA best reliable success predictor in nursing and other healthcare professions. Assessing personal attributes by interview despite poor predictive reliability Use of personal statements to examine the reasons for applicants to enter the field
Ruth-Sahd 2013, USA [46]. Review article	A review of the challenges facing nursing and medical curricular including admission requirements; suggestions about improving admission methods and teaching strategies.	Not applicable	Literature review	A minimum GPA requirement for entry to nursing school Scholastic Achievement Test (SAT) American College Test (ACT) Recommendation letters Written essays
Perkins et al. 2013, UK [47]. Research article	How effective is Multiple Mini Interviews al as a selection tool for entry into a nursing pro-gramme.	Assessment of St. George’s university 890 applicants and 82 Interviewers	descriptive design	More than 90% of participants preferred the MMI method, 65% preferred the MMI method over traditional interviews. The predictive validity of the MMI method is greater than that of traditional interviews.
Usher et al. 2013, Australia [48]. Research article	o explore the motivations of student nurses enrolled in nursing courses	152 nursing students	qualitative descriptive design	to improve recruitment strategies in the future by assessing the applicants’ personal characteristics, such as helping others (Reduce the suffering of the people, educating people about the disease, care of people)
Lancia et al. 2013, Italiy [49]. Research article	To investigate the role in predicting nursing students’ academic success.	1006 BSN students (five cohorts), matriculated in consecutive academic years from 2004 to 2008	retrospective observational study	The upper-secondary diploma coursework grades, unlike the admission test score, correlates positively with final degree grades and GPA of exam scores. Students who did not graduate within 6 semesters had lowest grades concerning their upper-secondary diploma coursework unlike the admission test score.
Lajoie 2013, USA [50]. Doctoral dissertation	To describe and compare reading comprehension of two groups of students, a pre-nursing student group and a senior nursing student group.	Two groups of students, a pre-nursing student group (*n* = 44) and a senior nursing student group (*n* = 44).	Descriptive design.	Pre-nursing and senior nursing students scored below the standardization norms for comparable college students, and senior nursing students also scored below the standardization values for other health profession students at a comparable level of education.
Underwood et al. 2013, USA [51]. Research article	To evaluate the use of HESI Admission Assessment (A2) exam as a predictor of student success.	184 BSN students.	Design not stated	HESI scores predicted the final course grades in all of the three first-semester nursing courses. As the HESI scores increased, so did the final course grades.
Taylor et al. 2014, UK [52]. Research article	Explore the literature regarding the efficacy, reliability and validity of face to face interviewing and related selection processes as selection tools Ascertain the views/perceptions of key stakeholders in relation to the selection process	7 higher institutions of higher education in Scotland with students, administration and clinical interviews participating.	mixed methods	Lack of research evidence regarding the validity and reliability of student selection methods, especially interviews Disagreement about the characteristics of applicants to enter the field assessing the non-cognitive and academic characteristics of applicants with different approaches (MMI, Personal statements, motivational letters, Literacy and numerical tests, Academic qualification, Personal and group interviews)
Jones-Schenk & Harper. 2014, USA [53]. Research article	To determine if students whose emotional intelligence characteristics meet or exceed those of successful staff nurses are more likely to be successful in a baccalaureate nursing program.	116 potential nursing students and 42 successful staff nurses	descriptive, correlational design	Students with higher levels of emotional intelligence, particularly intrapersonal capacity and stress tolerance, are more likely to be successful in a baccalaureate nursing program than students with lower levels.
Waugh et al. 2014, UK [54]. Research article	To identify potential attributes and key skills for entering the field of nursing and midwifery	502 participants	survey	Consensus in the top seven ranked attributes: honesty and trustworthiness, communication skills, being a good listener, patience and tactfulness, sensitivity and compassion, good team worker and the ability to seek and act on guidance.
Bremner et al. 2014, USA [55] Research article	To identify students most likely to succeed in nursing studies using TEAS	511 first semester students enrolled from fall 2011 to fall 2013	A cross-sectional, descriptive study	Test of Essential Academic Skills (TEAS) scores predicted first semester ATI proficiency
Harner 2014, USA [56]. Doctoral dissertation	To examine the relationship between TEAS scores and early academic success in a BSN program	218 nursing students.	correlational study	Two subcomponents of TEAS, namely Reading and English, were predictors of success in the first semester courses.
Hinderer et al. 2014, USA [57] Doctoral dissertation	To explore the HESI admission scores, preadmission cumulative GPA and science GPA as predictors of progression to nursing major and first-time success on the NCLEX-RN.	89 nursing students admitted 2008–2010 (three cohorts)	exploratory retrospective descriptive design	Health Education Systems, Inc. (HESI) score was correlated with nursing GPA and NCLEX-RN success but not with timely progression.
Sanneh & Mbuiya. 2015, Finland [58]. Master thesis	Outline the currently used student selection methods in nursing education and other healthcare professions and identify any existing relationship between these methods and education outcomes.	17 articles	Literature Review	GPA as the most recurring student selection method in nursing and other healthcare professions. Other selection methods include Multiple Mini Interview, Assessment centers, standardized preadmission tests Relationships between these methods and education outcomes have also been covered.
Pitt et al. 2015, Australia [59]. Research article	To explore entry critical thinking scores (Health Sciences Reasoning Test) in relation to demographic characteristics, students’ performance and progression	134 BSN students.	Longitudinal correlational study	Statistically significant relationship was established between students’ entry critical thinking scores, academic performance and ability to complete the program in 3 years. The strongest predictor of academic failure was students’ entry HSRT-test subscale scores. Critical thinking scores had no significant relationship to clinical performance.
Elkins 2015, USA [60]. Research article	To investigate the possible predictors of success in completing the baccalaureate nursing program and passing the NCLEX-RN licensure exam.	187 BSN nursing students from two courses admitted during fall 2007 and 2008.	Correlational study	A statistically significant relationship was identified between the preprogram GPA, ACT scores, anatomy grades, and the HESI Exit Exam scores with the completion of the BSN program and passing the NCLEX-RN.
Crouch 2015, USA [61]. Research article	To assess Watson-Glaser Critical Thinking Appraisal (WGCTA), prerequisite GPA and the National League of Nursing (NLN) preadmission test as a pre-admission criterion.	192 first-year nursing students.	Correlational study	WGCTA, prerequisite GPA and NLN had a statistically significant relationship with the nursing GPA. Strongest relationship between prerequisite GPA and the nursing GPA
MacDuff et al. 2016, UK [62]. Research article	To interpret perspectives regarding on-site selection of student nurses and midwives.	72 nursing students, 36 lecturers and 5 members of clinical staff from 7 Scottish universities	qualitative descriptive design	Staff used a range of attributes (interpersonal skills, team-working, confidence, problem-solving, aptitude for caring, motivations, commitment) as part of holistic assessments.
Wambuguh et al. 2016, USA [13]. Research article	Report on the Predictability of Current Admission Criteria for Nursing Program Success	513 students	descriptive, correlational design	Findings of this study highlight pre-admission TEAS scores and pre-admit science GPAs as the academic factors that are useful for the selection of students with a higher likelihood of success in nursing school programs, as defined by program completion, graduating with a nursing program GPA of 3.25 or higher, and passing the NCLEX-RN
Gale et al. 2016, UK [6]. Research article	To ascertain evidence of bias in Multiple Mini Interviews (MMI), and to determine the predictive value of the MMI of academic success.	204 students who commenced studies in September 2011.	A longitudinal retrospective design	MMI and MMI numeracy marks appeared to significantly predict academic success (assessment marks). MMI literacy results predicted weakly academic success. MMI showed little or no evidence of bias (gender, age, nationality, location of secondary education).
Simelane 2017. Africa [63]. Master of thesis	to explore nurse educators’ perceptions of the current selection criteria and describe the criteria that they would recommend for better selection	19 participants	qualitative, exploratory and descriptive design	Necessary criteria for selecting a nursing student include: compassion, empathy, passion, intelligence, caring characteristics, an innate desire to help others, medical monitoring of applicants for chronic illness.
Callwood et al. 2017, UK [64]. Research article	To examine the reliability and predictive validity of MMIs using end of Year One practice outcomes of under-graduate pre-registration nursing, midwifery and paramedic students	227 student (nursing, midwifery, paramedic)	Cross-discipline cohort study	MMIs are reliable VBR tools which have predictive validity when a seven station model is used.
Callwood et al. 2018, UK [65]. Review article	to Identifying personal domains for Nursing Students Selection in MMI Method	46 article	narrative synthesis systematic review	Communication/ interpersonal skills/ written communication, Teamwork/ collaboration/ collegiality, Ethical & moral judgment/ academic integrity/ social justice/research ethics/disclosure of error, Critical thinking, Empathy/ emotional maturity, Honesty/ integrity, Self-awareness/ reflection, Problem solving, Respect for others (difference and diversity, privacy and dignity)/ Cultural competency, Compassion, Decision making.
Talma et al. 2018, Finland [66]. Research article	To compare the predictive value of two on-site selection methods used in nursing student selection, namely, psychological aptitude tests and literature-based exams	626 nursing students	cohort study	To assess cognitive and non-cognitive skills of nursing students is required. Two on-site selection methods are predictive of nursing students’ levels of knowledge and skills (psychological test), and study success (literature based exam) at the beginning of their studies. Future research should also focus on the admission/selection costs to universities
Haavisto et al. 2019, Finland. [67] Research article	To develop an evidence-based structure and content for the new nursing entrance examination.	3 focus group interviews (*n* = 26) and 39 articles	interpretive descriptive design	Learning skills (Language and communication skills, Mathematical skills, reasoning skills, Information technology skills, Self-directed skills), Social skills (Ethicality, Interpersonal Communication, Emotional intelligence), Certainty of career choice (Realistic perception of nursing profession, Desire to work in nursing, Characterizing self as a nurse, Imaging nursing as an ideal career)
Yousafzai & Jamil 2019, Pakistan [68] Research article	To determine the relationship between various variables in the existing admission criteria and academic performance.	197 participants	cross sectional study	previous academic scores at diploma level were better predictors of the academic performance
McNeill et al. 2019, Canada [69]. Research article	Developing nurse match: A selection tool for evoking and scoring an applicant’s nursing values and attributes	63 first year nursing students	case study-based qualitative process	Person Centeredness, Accountability, Trust, Integrity, Commitment to Personal Development, Teamwork

## Results

### Literature search

A total of 5417 articles were found from databases search, duplicate articles were removed, and 3045 articles entered the title and abstract review phase. After excluding unrelated studies, the full text of 182 articles were evaluated in terms of inclusion criteria and 44 articles were included in the final review.

### Study characteristics

Most studies (*n* = 20) were from the USA followed by the UK (*n* = 9), Australia (*n* = 4), Finland (*n* = 3), Canada (*n* = 3) and one study from each of the countries New Zealand, Pakistan, Oman, Sweden, Africa, and Italy. Thirty-two articles were research studies, 4 were review articles and 8 were thesis and doctoral dissertations. Study characteristics are presented in Table 1.

### Nursing students’ selection criteria

The Review identified that nursing students are selected based on two criteria: (1) cognitive-academic abilities and (2) non-cognitive abilities. These two criteria are explained below.

### Cognitive-academic abilities

Most studies considered cognitive-academic abilities as an essential criterion for nursing student admission. The four most common cognitive-academic competencies evaluated in nursing applicants included (1) reasoning skills (analysis ability, deductive and inductive reasoning, inference, critical thinking, problem-solving, decision-making evaluation, logic); (2) mathematical skills (math, numeracy, basic calculation, applied math); (3) language skills (English writing, reading comprehension, reading, vocabulary, English reading, general knowledge of the language, word knowledge, literacy, verbal); and (4) natural science skills (chemistry, physics, biology, anatomy and physiology). Nursing applicants were assessed for language and mathematical skills in the majority of studies, and few studies focused on assessing reasoning and natural science skills of nursing applicants (Tables 1 and 3).

### Non-cognitive abilities

Reviewed studies revealed that non-cognitive abilities examined in nursing applicants include communication skills, teamwork, dynamism, morality, psychological strength, Emotional intelligence and warmth (As seen in Table 2).

**Table 2:** Non-cognitive abilities used in the selection process for nursing students

Categories	Definition	Factors	Relevant Studies
Communication skills	Collect and convey information in order to create and sustain relationships with others in appropriate manner.	appropriate non-verbal communication/body language	Taylor R et al., 2014 [52], Waugh A et al., 2014 [54], Gale J et al., 2016 [6], Haavisto E et al., 2019 [67], Perkins et al., 2013 [47], Callwood A et al., 2018 [65]
		active listening	
		expressiveness	
		Engages in social conversation	
		Able to give advice, and give directions to others	
Teamwork	Effectively and respectfully work with others	cooperativeness,	Gale J et al., 2016 [6], Callwood A et al., 2018 [65], Perkins et al., 2013 [47], Taylor R et al., 2014 [52], McNeill C et al., 2019 [69], Waugh A et al., 2014 [54]
		collegiality	
		Ability to work closely with others	
Dynamism	Seek for learning opportunities, Flexibility to change and Being challenging	open minded	Haavisto E et al., 2019 [67], McNeill C et al., 2019 [69], Jones-Schenk & Harper, 2014 [53]
		Self-directed skills	
		Commitment to Personal Development	
		adapt to an environment that may change rapidly	
		Not being resistant to change,	
		adaptability	
Morality	To act in accordance with ethical principles and standards of conduct	ethical insights (ethical decision making, moral judgment)	Gale J et al., 2016 [6], Haavisto E et al., 2019 [67], Callwood A et al., 2018 [65], Jones-Schenk & Harper, 2014 [53], McNeill C et al., 2019 [69], Waugh A et al., 2014 [54], Taylor R et al., 2014 [52]
		Responsible	
		Conscientious	
		Accountability	
		Reliable	
		Trustworthy	
		Honesty	
		disclosure e of error	
		integrity	
		Respect for others (privacy and dignity)	
Psychological strength	Ability to deal with the trials and tribulations	stress management,	Jones-Schenk & Harper, 2014 [53], Waugh A et al., 2014 [54]
		tolerance highly stressful situations	
		able to stand the sight of blood, able to cope with death	
		Patient	
Emotional intelligence	Accurately recognize and understand one’s own emotions and those of others, using this information to guide future behavior.	emotion perception (understanding emotions, Understanding and supportive)	Haavisto E et al., 2019 [67], Gale J et al., 2016 [6], Callwood A et al., 2018 [65], Taylor R et al., 2014 [52], Waugh A et al., 2014 [54]
		Understand and control reactions to the behaviors and emotions of others	
		emotional maturity	
		Sensitive to others and self	
		Able to give advice, and give directions to others	
Warmth	Demonstrate affection or enthusiasm in behavior.	Kindness, friendly	Gale J et al., 2016 [6], Ruth Sampie Simelane 2017 [63], Callwood A et al., 2018 [65], Waugh A et al., 2014 [54], Jones-Schenk & Harper, 2014 [53], Pitt V et al., 2012 [42]
		Compassionate	
		Altruistic, (Desire to help, Inherent desire to care)	
		Empathy	

### Methods used to assess nursing student selection criteria

Results of the review indicated that two main methods are used to assess the cognitive-academic competencies of nursing applicants are:

1. On-site test for selection (conducted either before or during the Student selection process): According to the reviewed studies, standardized tests are often used to measure cognitive-academic abilities in this method (Table 3).
2. Academic achievement records: In most studies, academic records have been used as the most common criterion for selecting a student for nursing education, typically based on the high school grade point average (GPA) [8, 13, 14, 32, 35, 37, 45, 46, 49, 52, 58, 61, 68]. Studies have reported prior academic achievement of applicants in general, but it was not possible to further analyze the specific cognitive-academic abilities acquired from academic records of applicants.

**Table 3:** Onsite selection methods of assessing cognitive-academic abilities

Name of type of the selection/developer	Items
**Standardized tests**	
SAT (Scholastic Aptitude Test) Grossbach & Kuncel 2011 [41], Jarmulowicz 2012 [43], Stuenkel 2006 [30], McGahee, Gramling and Reid 2010 [35]	Verbal, math
ACT (American College Test) Elkins 2015 [60], Grossbach and Kuncel 2011 [41], Jarmulowicz 2012 [43], McGahee et al. 2010 [35]	English (reading, writing), math, natural science, social science
TEAS (Test of Essential Academic Skills) Bremner et al. 2014 [55], Harner 2014 [56], Hernandez 2011 [39], Newton & Moore 2009 [34], Newton et al. 2007 [31], Wolkowitz & Kelley 2010 [36]	Reading, mathematics, science (life science, earth science, physical science, human body science), and English language usage
HESI (Health Education Systems Inc) Hinderer et al. 2014 [57], Underwood et al. 2013 [51]	English: reading comprehension, vocabulary & general knowledge, grammar. Math: Basic math skills. Science: biology, chemistry, anatomy& physiology, physics
HSRT (Health Sciences Reasoning Test) Pitt et al. 2015 [59]	Total critical thinking skills, analysis, inference, evaluation, deductive reasoning and inductive reasoning.
NLN (National League for Nursing) Crouch 2015 [61], Stuenkel 2006 [30]	Not stated in the articles. From NLN website (2017): Verbal–Word knowledge and reading comprehension. Math –Basic calculations, word problems, applied math. Science–General biology, chemistry, physics and earth science
NET (Nurse Entrance Test) Herrera 2012 [44]	Math skills, reading comprehension
NDRT (Nelson-Denny Reading Test) Lajoie 2013 [50]	Vocabulary, reading comprehension, reading rate.
WGCTA (Watson-Glaser Critical Thinking Appraisal) Crouch 2015 [61]	Critical thinking
**Other selection methods**	
Literacy and numeracy test MacDuff et al. 2016 [62]	Literacy and numeracy skills
MMI (Multiple Mini Interview) Gale et al. 2016 [6], MacDuff et al. 2016 [62], Perkins et al. 2013 [47], Timer & Clauson 2011 [8]	Cognitive attributes: numeracy skills, literacy skill, decision-making skills, problem-solving skills
Nationwide Entry Exam Dante et al. 2011 [40], Lancia et al. 2013 [49]	General education, mathematics, logic, biology, chemistry, physics
Onsite student selection processes: Interview MacDuff et al. 2016 [62]	Cognitive attributes: problem-solving

Based on the review results, the TEAS was the most commonly used test, yet reliability of test was only confirmed in one study (NDRT test: Nelson-Denny Reading Test) [50]. The reliability or validity of other selection tests reported based on previous assessments by instrument developers in the studies [36, 39, 47, 51, 55, 59, 61]. In other studies, the reliability and validity of the test used was not reported [30, 31, 34, 40, 41, 43, 49, 56, 57, 60].

Four main methods were found to assess the non-cognitive abilities of nursing applicants. Interviews (panel interviews or multiple mini interviews) are the main method used to assess communication skills, teamwork morale, ethical insights, and empathy. Personal statements were another selection method, commonly used to assess motivation and self-assessment of personal characteristics. Some nursing institutes also use recommendation letters provided by teachers and there was limited used of personality tests (Table 1).

### Methods of student selection and relationship with academic performance

The relationship of selection methods and academic performance was reported positive in 20 articles and neither positive nor negative in 5 articles. The relationship of academic performance with standardized tests (15 articles) and academic records (13 articles) has been examined more than other methods of student selection. Only two articles reported a positive relationship between interviews (individual interview and multiple mini interviews) and academic performance. In most studies, academic success and passing the NCLEX exam (National Council Licensure Examination) have been used as a criterion for assessing academic performance. The relationship between the selection methods (i.e. HSRT: Health Sciences Reasoning Test) and clinical performance has been examined in only one study without identifying a positive or negative relationship (Table 4).

**Table 4:** The Relationship between student selection methods and academic performance in reviewed studies

Selection methods	Author and years	***P***-value for relationship of student selection methods to academic performance				
		Academic success	Attrition	Graduation	NCLEX-RN	Clinical performance
American College Test (ACT)	Elkins 2015 [60]				<.05a	
	Grossbach & Kuncel 2011 [41]				<.01a	
Health Education Systems Inc. (HESI) Admission	Hinderer et al. 2014 [57]	.007a		Not report	.01a	
	Underwood et al. 2013 [51]	<.01b				
Health Sciences Reasoning Test (HSRT)	Pitt et al. 2015 [59]	<.01a		<.01b		>.01b
National League for Nursing (NLN)	Crouch 2015 [61]	<.001a				
	Stuenkel 2006 [30]				<.001a	
Scholastic Aptitude Test (SAT)	Grossbach and Kuncel 2011 [41]				<.01a	
	Stuenkel 2006 [30]				<.001a	
Test of Essential Academic Skills (TEAS)	Bremner et al. 2014 [55]	<.001a				
	Harner 2014 [56]	<.001a				
	Hernandez 2011 [39]	<.001a	<.001a			
	Newton et al. 2007 [31]	<.001a				
	Wolkowitz & Kelley 2010 [36]	< 0.001b				
	Newton & Moore 2009 [34]		.329b			
	Wambuguh et al. 2016 [13]			.01b	.02b	
Watson-Glaser Critical Thinking Appraisal	Crouch 2015 [61]	<.01a				
Nurse Entrance Test (NET)	Herrera 2012 [44]			>.01b		
Nationwide Entry Exam	Dante et al. 2011 [40]	.006b		.001b		
	Lancia et al. 2013 [49]	.38a		.215a		
Previous academic achievement	Newton et al. 2007 [31]	<.001b				
	Newton & Moore 2009 [34]	<.001a				
	Lancia et al. 2013 [49]	.001a		.001a		
	Crouch 2015 [61]	< .01a				
	Timer & Clauson 2011 [8]	<.001b				
	Wambuguh et al. 2016 [13]	.001b		.01a		
	Elkins 2015 [60]			<.01a	<.01a	
	Herrera 2012 [44]			<.001a		
	Schmidt & MacWilliams 2011 [37]			<.01a	<.01a	
	Hernandez 2011 [39]			<.01a	<.01a	
	Grossbach & Kuncel 2011 [41]				<.01a	
	Stuenkel 2006 [30]				<.01a	
	McGahee et al. 2010 [35]				.002a	
Interviews	Gale et al. 2016 [6]	.03b				
	Schmidt & MacWilliams 2011 [37]		<.01b			

a = Pearson correlation coefficient, b = regression analysis

*NCLEX-RN* National Council Licensure Examination-Registered Nurse

## Discussion

This study assessed existing published literature on the admission criteria and selection methods of undergraduate nursing students. Results showed that academic-cognitive and non-cognitive abilities are the main two criteria in the process of selecting students for nursing programs. According to the results of this review, the academic-cognitive abilities of the applicants are mainly examined through the academic records and standardized tests, and the non-cognitive abilities are investigated through the interviews, personal statements and references.

Review of the selected studies showed that academic abilities of applicants are assessed in three main areas of mathematics, language and natural sciences skills which aligns with the World Health Organization recommendations for selection criteria in nursing students [26]. Basic science skills were suggested in previous studies without any complete explanation. In this study, the most important basic science skills were identified. According to the results of this review, academic abilities are good predictors of academic success of nursing students [8, 39, 40, 51, 56, 57, 61].

Cognitive abilities were another criterion for selecting the nursing student in the reviewed studies. Although the cognitive abilities are very important for all students of the higher education institutions [70], however, the investigation of this criterion among the nursing applicants is of special importance [67]. Cognitive abilities are very crucial in the complex working environments, including the nursing [70]. The nursing field is complex and the undergraduate students must acquire the necessary qualifications for nursing in a relatively short period of time [71]. Therefore, the cognitive preparation is necessary for the individuals to succeed in the theoretical and clinical courses [72]. The research findings also indicate that the nursing applicants who have been investigated according to the reasoning skills have the theoretical and clinical success during their training [59]. The nurses’ cognitive abilities play a key role in the problem-solving skills, the clinical decision-making power, and as a results diagnosing the patient needs and selecting the best nursing practices [73, 74]. This could directly affect the patient’s safety and improvement [75]. However, the results of this study showed that cognitive abilities of applicants have been assessed in few articles. In this regard, the European Federation of Nurses Association has acknowledged that although this skill is considered an important competence in nursing education, it is usually neglected and under-valued when selecting nursing students [76]. These findings demonstrate the need for assessing reasoning skills for selecting nursing students.

The results of this study showed that the cognitive-academic abilities of applicants are assessed mainly through academic records or standardized tests [37, 46]. In order to evaluate this ability, the research evidence suggests that the standardized tests and academic records are more relevant to the future academic performance of the nursing students than the other methods (interview and non-standardized tests) [6, 30, 36, 51, 55, 57, 59, 61], and are better predictors of nursing students’ academic success [30, 39]. However, the findings of this study indicated that none of the standardized tests evaluate all of the four cognitive-academic skills in one test. On the other hand, there is little research evidence on the validity and reliability of nursing standardized tests [30, 31, 34, 46, 49, 51, 56–58, 60, 61]. In addition, the most important criticism of using academic records as a selection criterion is heterogeneity of scores, since they are obtained from different institutions, leading to bias in the selection of nursing students [8]. It is worth mentioning that academic records can be a good criterion for students’ selection provided that valid standardized tests are nationally conducted.

The non-cognitive skills were another criterion for selecting the nursing student in the reviewed studies. It is important to select nursing students with non-cognitive, professionally tailored characteristics to provide safe and high quality care [77]. According to research findings, traits such as empathy and morality of nursing students do not change during their training which highlights the importance of their assessment when entering the nursing profession [78]. Researchers have concluded that academic-cognitive abilities are necessary but not sufficient for becoming a qualified nurse and this criterion alone cannot guarantee ethical and appropriate practice in nursing [66]. Individual values, interests and motivations are not considered in this approach, and individuals with high academic-cognitive abilities cannot be considered competent and qualified nurses merely through education [66]. According to Ones et al., cognitive abilities along with non-cognitive abilities lead to better performance of an individual in a job [79]. Therefore, non-cognitive characteristics should be considered a key criterion in nursing student’s selection [8, 66].

This review indicates that assessment of non-cognitive abilities is generally done through interview (traditional, multiple mini interview), personal statements, references and personality assessmentt [8, 32, 37, 45, 47]. Interviews are the most common method for assessing non-cognitive abilities such as communication and teamwork skills [32, 37, 45, 47, 52, 58], despite evidence that traditional interviews lack predictive validity and are not a powerful tool for selecting nursing students [8, 45, 80]. Interviews are strongly influenced by interviewers [81] and hence are highly associated with bias in the selection process [37]. More recently, some universities have begun using multiple mini interviews to select applicants [47], which have been found to have higher validity and reliability compared to traditional interviews [47, 58]. However, limited studies exist on the predictive validity of MMI [6, 47]. Construct validity of MMI remains a challenge, and there is insufficient consensus on the dimensions that applicants need to be examined in multiple mini interviews and thus requires further research evidence [47, 52]. Multiple mini interview is also a costly method because it requires station design as well as more manpower and role players [47, 82, 83]. Personal statements are another method used to assess non-cognitive characteristics including motivation and self-evaluation [8, 45]. There is little research evidence to confirm the predictive validity of personal statements, and most research evidence indicates that this method lacks validity and reliability as a selection tool [8, 45, 46, 52]. On the other hand, the content of personal statements may lead to unfair judgment in the selection of applicants [84]. There is limited studies regarding the use of references as a student selection method and their use is not recommended due to low reliability and validity [8, 46, 52]. Despite these findings, most nursing schools widely use personal statements and references for student selection. Some studies have suggested personality assessment to assess non-cognitive abilities. The results of a Meta-analysis on the predictive validity of personality assessment showed an insignificant relationship between personality predictors and job criteria [79]. Despite low validity, these tests have been widely used in selecting health care professionals for many years [85].

In addition to the above-mentioned methods, selection centers and situational judgment tests are also used for assessing the non-cognitive abilities suggested for medical students. Research evidence regarding the use of selection centers for selecting medical applicants indicates high validity of this method, but it can be costly for institutions [86, 87]. Situational judgment tests have also been recognized as a reliable valid method for assessing non-cognitive abilities and are used to examine a wide range of non-cognitive traits for selecting many large-scale job applicants [88, 89]. Despite the use of situational judgment tests for student selection in some health care professions [90–94], no research evidence was found regarding the use of this method for nursing student selection.

### Limitations

The findings of this scoping review must be interpreted with caution because the quality of the selected articles was not evaluated. Therefore, articles of varying quality were included in this study and the results may be of limited reliability.

## Conclusion

The results of this scoping review can be used by nursing education policymakers and institutes for comprehensive assessment of applicants in terms of their suitability for the nursing education. Both academic-cognitive and non-cognitive abilities should be considered when selecting a student for entry into nursing education. Future studies should be directed toward assessing and improving methods of student selection. According to the reviewed studies, there is limited evidence on content and predictive validity of selection methods including MMIs and standardized tests. Longitudinal studies (examining students during the course of study and career) are required to assess predictive validity of these methods. The findings of this review showed insufficient consensus among researchers about which non-cognitive characteristics should be examined in nursing applicants. Further research is required to identify attributes considered essential for success during nursing training and nursing practice. The relative contribution of each selection criterion in the student admission system is also unclear; therefore, further research is needed to weigh the selection criteria. Given the lack of research evidence on the situational judgment tests in nursing education despite its cost-effectiveness and large-scale feasibility, it is suggested to design these tests to examine the non-cognitive characteristics of applicants.

## Supplementary Information

**Additional file 1.** Preferred Reporting Items for Systematic reviews and Meta-Analyses extension for Scoping Reviews (PRISMA-ScR) Checklist.

## Data Availability

The datasets used and/or analysed the current study are available from the corresponding author upon reasonable request.

## Abbreviations

GPA: Grade point average
NDRT: Nelson-Denny Reading Test
NCLEX: National Council Licensure Examination
ACT: American College Test
TEAS: Test of essential academic skills
HSRT: Health Sciences Reasoning Test
HESI: Health Education Systems Inc
MMI: Multiple mini interview
BSN: Bachelor of Science in Nursing
NLN: National League for Nursing
RN: Registered Nurse
NCEA: National Certificate of Educational Achievement
SAT: Scholastic Achievement Test
NET: Nurse Entrance Test
WGCTA: Watson-Glaser Critical Thinking Appraisal
